# Sexual Dimorphism in the Early Embryogenesis in Zebra Finches

**DOI:** 10.1371/journal.pone.0114625

**Published:** 2014-12-10

**Authors:** Makhsud Tagirov, Joanna Rutkowska

**Affiliations:** 1 Poultry Research Institute, Ukrainian Academy of Agrarian Sciences, Borky, Ukraine; 2 Institute of Environmental Sciences, Jagiellonian University, Kraków, Poland; Leibniz Institute for Age Research - Fritz Lipmann Institute (FLI), Germany

## Abstract

Sex-specific gene expression before the onset of gonadogensis has been documented in embryos of mammals and chickens. In several mammalian species, differences in gene expression are accompanied by faster growth of pre-implantation male embryos. Here we asked whether avian embryos before gonadal differentiation are also sex-dimorphic in size and what genes regulate their growth. We used captive zebra finches (*Taeniopygia guttata*) whose freshly laid eggs were artificially incubated for 36–40 hours. Analyses controlling for the exact time of incubation of 81 embryos revealed that males were larger than females in terms of Hamburger and Hamilton stage and number of somites. Expression of 15 genes involved in cell cycle regulation, growth, metabolic activity, steroidogenic pathway and stress modulation were measured using RT-PCR in 5 male and 5 female embryos incubated for exactly 36 h. We found that in the presence of equal levels of the growth hormone itself, the faster growth of male embryos is most likely achieved by the overexpression of the growth hormone receptor gene and three other genes responsible for cell cycle regulation and metabolism, all of them located on the Z chromosome. Autosomal genes did not show sex-specific expression, except for the steroidogenic factor 1 which was expressed only in female embryos. To our knowledge this is the first report of sexual size dimorphism before gonadogenesis in birds. The finding suggests that faster growth of early male embryos is conserved through the mammalian and bird phyla, irrespective of their differential sex chromosome systems.

## Introduction

For several decades it has been assumed that sex differences in phenotype appear only with the start of sex hormone secretion by the gonads [1]. However, recent data, provided mostly by the high throughput genomic research and analyses of gene expression and function, indicate that sex differences in gene expression are present at the very beginning of embryonic development [2], [3], [4], [5] and thus suggest that sexual dimorphism could precede differentiation of the gonads.

The phenomenon of faster male development at the pre-implantation stage was first detected in mouse embryos [6] and was later confirmed in all studied eutherian mammals: cows [7], [8], [9], sheep [10], mice [11], [12], [13], pigs [14] and humans [15], [16], [17]. The most noticeable features of mammalian male and female pre-implantation embryo differences are the faster rate of male growth and the higher viability of females (e.g. [3]). Differences in growth rate reflect differences in metabolic activity, which is higher in males. For instance, in the cow, total glucose metabolism is two-fold higher in males compared to females, and the activity of the pentose phosphate pathway is four times higher in females than in males [18]. The molecular mechanism behind those effects is disputable. One the one hand, the phenotypic differences between mammalian male and female embryos were suggested to be underlined by differential expression level of glucose 6-phosphate dehydrogenase (*G6PD*) and hypoxanthine phosphoribosyl transferase (*HPRT*) - two genes located on the X chromosome involved in energetic metabolism and modulation of cellular oxidative stress [19]. Their overexpression in female bovine pre-implantation embryos is thought to reduce the impact of oxygen radicals that stimulate growth (and thus lead to the smaller size) of female embryos while ensuring their higher viability [20]. On the other hand, some studies report cell proliferation and growth promoting role of the *G6PD* [21]. Overall, at the pre-implantation stage, genes located on the X chromosome are more likely to be overexpressed in females [2], but role of specific genes in shaping sexual size dimorphism at that stage has not been studied.

It is not known whether faster male development is universal to males of all animal species or is a by-product of the mammalian sex chromosome system. The study of sex differences in birds, taxa with a different sex chromosome profile and different evolutionary origin of the sex chromosomes compared to mammals (e.g. [22]), could provide valuable information about genetic mechanism and the (potential) importance of the phenomenon. Similar to findings in mammals, considerable sex differences in gene expression levels have been detected in birds [4], [5], [23]. Microarray analyses of the chicken genome showed that genes involved in fatty acid and carbohydrate metabolism and mitochondrial and cell cycle processes are expressed at higher rates in male than in female 1-day old embryos. Conversely, female embryos showed higher expression of genes involved in enzyme inhibition, negative control of cell division and epigenetic regulation [5], [24]. The vast majority of genes exhibiting sex-biased expression level in birds are located on the Z chromosome, but a small percentage of autosomal genes also show sex-specific expression in early embryos [5]. Importantly, all those differences are apparent before the onset of gonadal differentiation, which starts at stage 30 of the Hamburger and Hamilton [25], i.e. after 6.5–7 days of egg incubation. So far, no studies have investigated the variation in size in the avian embryo before gonadal differentiation.

Given what is known about sex-dimorphic gene expression profiles in early embryos of birds [5] and the sex dimorphism in development of pre-implantation mammalian embryos [6], [13], we hypothesize the existence of similar differences in the growth rate and transcriptional activity of early bird embryos. The aim of this paper is to investigate sex differences in growth rate and in expression of genes underlying size differences in 36 h zebra finch embryos. Specifically, we first tested the prediction of existence of morphological differences between two sexes long before the start of sex hormone secretion. Next, we tested the prediction that the higher growth rate should be supported by the overexpression of the genes involved in the positive control of cell cycle and embryo growth. We analyzed the relative expression profiles of genes generally involved in cell growth and differentiation, namely cell proliferation promoting genes, genes from the growth hormone (GH) pathway, key metabolic, stress response and steroidogenic genes.

## Materials and Methods

### Ethics statement

The study was performed according to the agreement from the First Local Ethical Committee on Animal Testing at the Jagiellonian University in Kraków (decision number 53/2012).

### Isolation of embryos

Zebra finches (*Taeniopygia guttata*) from the captive colony maintained in the Institute of Environmental Sciences of the Jagiellonian University, were used for the experiments. Twenty breeding pairs housed in separate cages produced on average 4.05 eggs. The nest boxes were checked for newly laid eggs every 2 hours after the lights in their room were switched on. Collected eggs were artificially incubated at 37°C in a climatic chamber (Binder, Germany). Following incubation, the eggs were cooled to stop their development. After that, the yolk ball was insulated in a 60 mm Petri dish with phosphate buffered saline (PBS), and cleaned from the albumen. The vitelline membrane was cut around the blastoderm. Gentle pipetting with the embryo transfer pipette allowed the embryo to detach from the vitelline membrane into the PBS solution. The embryo was washed in two changes of the solution and used for photography and downstream analyses.

### Morphological sex dimorphism in the early zebra finch embryos

**Figure 1:**
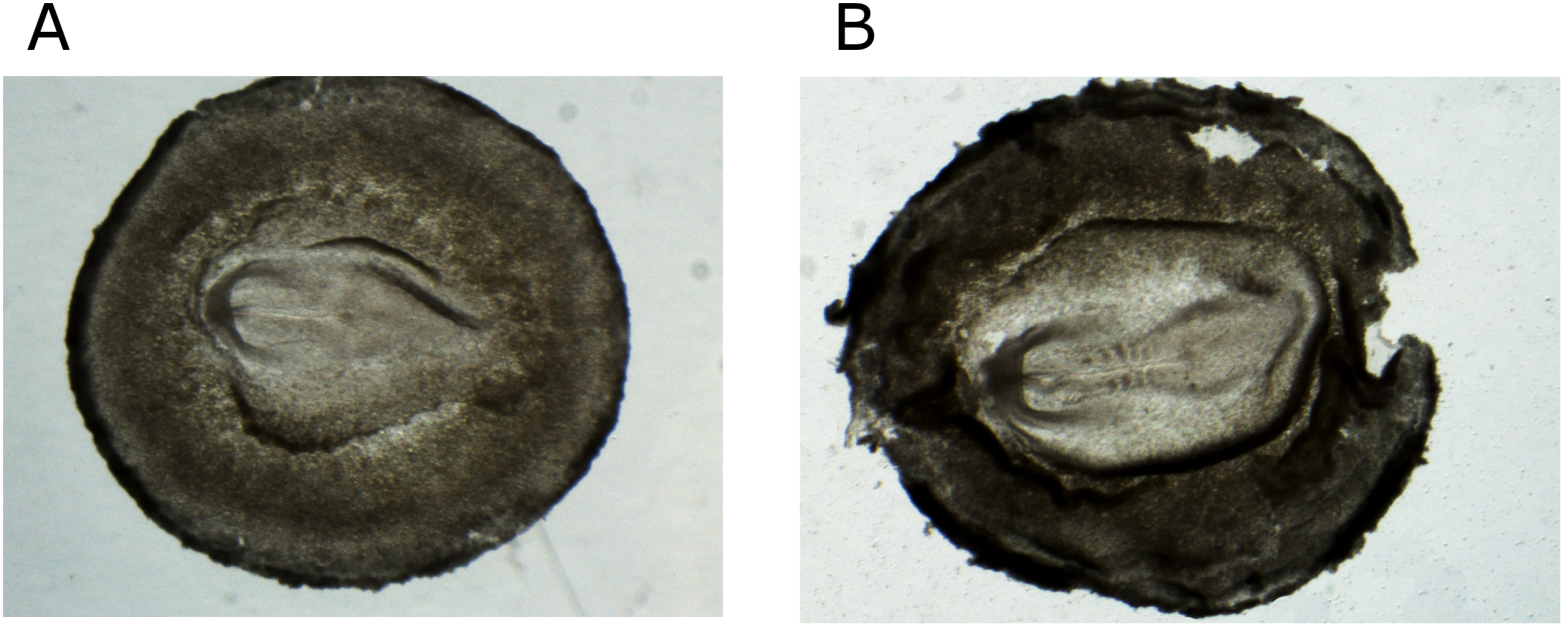
Representative photomicrographs of the zebra finch embryos after 36 h of egg incubation. A – embryo at 6th stage of H&H. There are no visible signs of somite formation and hardly any head fold. B – embryo at 8th stage H&H of development. At that stage the typical embryo has 4 to 5 pairs of somites and well developed head folder.

For detection of morphological differences between the sexes, the eggs were incubated for 36 h (N = 62), 37 h (N = 8) and 40 h (N = 11). The morphology of the incubated embryos was analyzed under the stereomicroscope (SZX7, Olympus, Japan) and staged using Hamburger and Hamilton scale (Fig. 1, [26], [27]). Subsequently a small piece of the embryo tissue was taken to isolate the genomic DNA for PCR amplification and embryo sex identification.

### Embryo sex determination

For embryo sex determination, two pairs of primers (Z1, Z2 and W1, W2) specifically designed for amplification of fragments of the sex chromosome in zebra finches [28] were used. The primers produce a single Z-band in males, and Z- and W-bands in females. PCR amplifications were carried out in 10 µl reactions containing 1x PCR buffer with (NH_4_)_2_SO_4_, MgCl_2_ (2.5 mM), dNTP mix (0.2 mM each), Dream Taq DNA polymerase (0.33 U/10 µl, Thermo Scientific, USA), each primer (0.5 µM), and 1 µl of gDNA (25–100 ng). Water was added to total reaction volume 10 µl.

PCR amplification was performed in a PCR Thermocycler Mastercycler Gradient (Eppendorf, Germany) in the following steps: denaturation at 95°C for 1.5 min; 35 cycles (95°C for 45 sec, 52°C for 45 sec, 72°C for 45 sec); and final extension at 72°C for 5 min. PCR products were separated on a 3% agarose gel stained with GelRed (Sigma Aldrich, Germany) and visualized using a UV light transilluminator.

### DNA and RNA isolation and gene expression analysis

To study the underlying genetic background of the observed differences in embryonic growth, we analyzed expression of the following genes involved in cell cycle control: *CDK1*, *CDK7*, *MAPK12*, *SMAD2*, *PRKAA1*, *CCND1*, *E2F6*, *P21*; growth hormone pathway: *GH1*, *GHR* and *JAK2*; cell energetic metabolism: *H6PD* and *FBP1*; steroidogenic pathway: *NR5A1*, and stress response: *NR3C1* and *NR3C2*.

Extraction of embryonic genomic DNA or total RNA were performed using AllPrep DNA/RNA Micro Kit (Qiagen, USA) following manufacturer protocols. The total DNA and RNA concentrations were quantified by Nanodrop 1000 spectrophotometer (Biotechnologie, GmbH). We attempted to isolate RNA for qPCR from the embryos after 6, 12, 20 and 36 h of incubation. The majority of embryos incubated for less than 36 hours yielded RNA of low quality and quantity. Thus, further analyses were performed on high quality RNA obtained from embryos incubated for 36 h. Five male and five female embryos were used for testing each gene. Equal amounts of RNA from male and female samples were added into the reaction for cDNA synthesis with AccuScript High Fidelity 1-st Strand cDNA Synthesis Kit (Agilent Technologies, USA). The reaction of reverse transcription (RT) was primed by random 15-mers in 20 µl of total reaction volume. In addition, in 3 embryos incubated for 6 hours we used gene specific primers to amplify cDNA of the growth hormone gene.

After the RT reaction, 1 µl of each cDNA product was mixed with the analyzed gene detection primers (Table 1) designed using the publicly available “Primer3” software (http://bioinfo.ut.ee/primer3) and iQ SYBR Green Supermix (Biorad, USA) in the total volume of 10 µl. Glyceraldehyde-3-phosphate dehydrogenase (GAPDH) and β-actin genes were selected as the reference genes. Real Time PCR was run in the 7500 Fast Real Time PCR System (Applied Biosystems). The thermo cycling conditions were as follows: 95°C for 3 min; 40 cycles of 95°C for 15 sec, 60°C for 30 sec, 72°C for 30 sec. The specificity of amplification during RT- PCR was confirmed by checking the melting curve. All reactions were run in duplicates.

**Table 1:** Pairs of primers used in the study.

Cell cycle control genes		
*SMAD2*	F	5′AGGGGAGAGGAGGAAGAAGG3′
*SMAD2*	R	5′CGGAGGAGTGAATGGCAATA3′
*CDK7*	F	5′GTGCAGCTGGTGATGATCTG3′
*CDK7*	R	5′GCAGCTGATTTCCTGGAGTG3′
*CCND1*	F	5′CTCCTACTTCAAGTGCGTGC3′
*CCND1*	R	5′ACTTCCTCTTCGCACTTCTG3′
*PRKAA1*	F	5′CGGTGGCGGATAAACAGAAG3′
*PRKAA1*	R	5′TCAGGATCTTCACGGCAACT3′
*E2F6*	F	5′TCAGCCATGGAAGAAGCTCT3′
*E2F6*	R	5′TCCTGAAATGCCTGAATGCT3′
*MAPK12*	F	5′GGCAAGGCATACAGACAGTG3′
*MAPK12*	R	5′TACAGCCCACAGACCAGATG3′
*CDK1*	F	5′CAGCCTCGAAACACATGTCA3′
*CDK1*	R	5′GCAGGAAGAGTGGATTTGTCC3′
*P21*	F	5′CTGCCTCCCAATCCAGAAGA3′
*P21*	R	5′AGTCTGAAGGGAGGGAGATCT3′
**Growth hormone pathway genes**		
*GHR*	F	5′ATCCACCACCAACAGCAGAT3′
*GHR*	R	5′ACCATTGTTGAGAGCCTGG3′
*GH*	F	5′GTCAAGCAACACCTGAGCAA3′
*GH*	R	5′ACAGCGATGAGGAGAGGAGA3′
*JAK2* *JAK2*	FR	5′CCTAAAGAGTAACAAGAAGCTGC3′5′TCCACGGATGTCACCATTCT3′
**Cell energy metabolism genes**		
*H6PD*	F	5′GAGTTTCCAGCTGCTTTTGC3′
*H6PD*	R	5′CTCAGCATCCTGGGAGAGAG3′
*FBP1*	F	5′ACAAAGATGCTGTGATAGTGGA3′
*FBP1*	R	5′CAGAAGGTTCATTAGGGGACAC3′
**Stress response genes**		
*NR3C1*	F	5′TGGAATAGGTGCCAGGGATC3′
*NR3C1*	R	5′GGTTGTGGATGGAGAAGAGC3′
*NR3C2*	F	5′TCATTCGGAGTTGGCATCCA3′
*NR3C2*	R	5′TCTCCACACACCAAACACAC3′
**Steroidogenic gene**		
*NR5A1*	F	5′CACGGAGCTGCATCTTCTTC3′
*NR5A1*	R	5′AGCTGTCGGTAGATGTGGTC3′
**Housekeeping genes**		
*β-actin*	F	5′CTGGCACCACTCCTTCTACA3′
*β-actin*	R	5′ATACATGGCTGGGGTGTTGA3′
*GAPDH*	F	5′CGAAGCGGTAAAGATGGTGA3′
*GAPDH*	R	5′ATGAAGGGATCGTTGATGGC3′
**Sex chromosome specific primers**		
W1	F	5′GGGTTTTGACTGACTAACTGATT3′
W2	R	5′GTTCAAAGCTACATGAATAAACA3′
Z1	F	5′GTGTAGTCCGCTGCTTTTGG3′
Z2	R	5′GTTCGTGGTCTTCCACGTTT3′

### Statistical analysis

To analyze variation in the size of embryos (in terms of H&H stage and number of somites), we used general linear mixed model in which embryo sex and duration of incubation were fixed factors and maternal identity was a random effect. The interaction between the fixed factors was not significant and was therefore removed from the final models. Those analyses were performed in SAS. The RT-PCR data of gene expression levels were analyzed using publicly available software Relative Expression Software Tool “REST” [29]. The software uses a pair-wise fixed reallocation randomization test, which repeatedly and randomly reallocates the observed Ct values at least 2000 times and compares male to female focal gene expression level taking into account the expression of the housekeeping genes. In all analyses, the tests were two-tailed and the level of significance was p<0.05.

## Results

### Sex dimorphism in the growth rate of the zebra finch embryos

**Figure 2:**
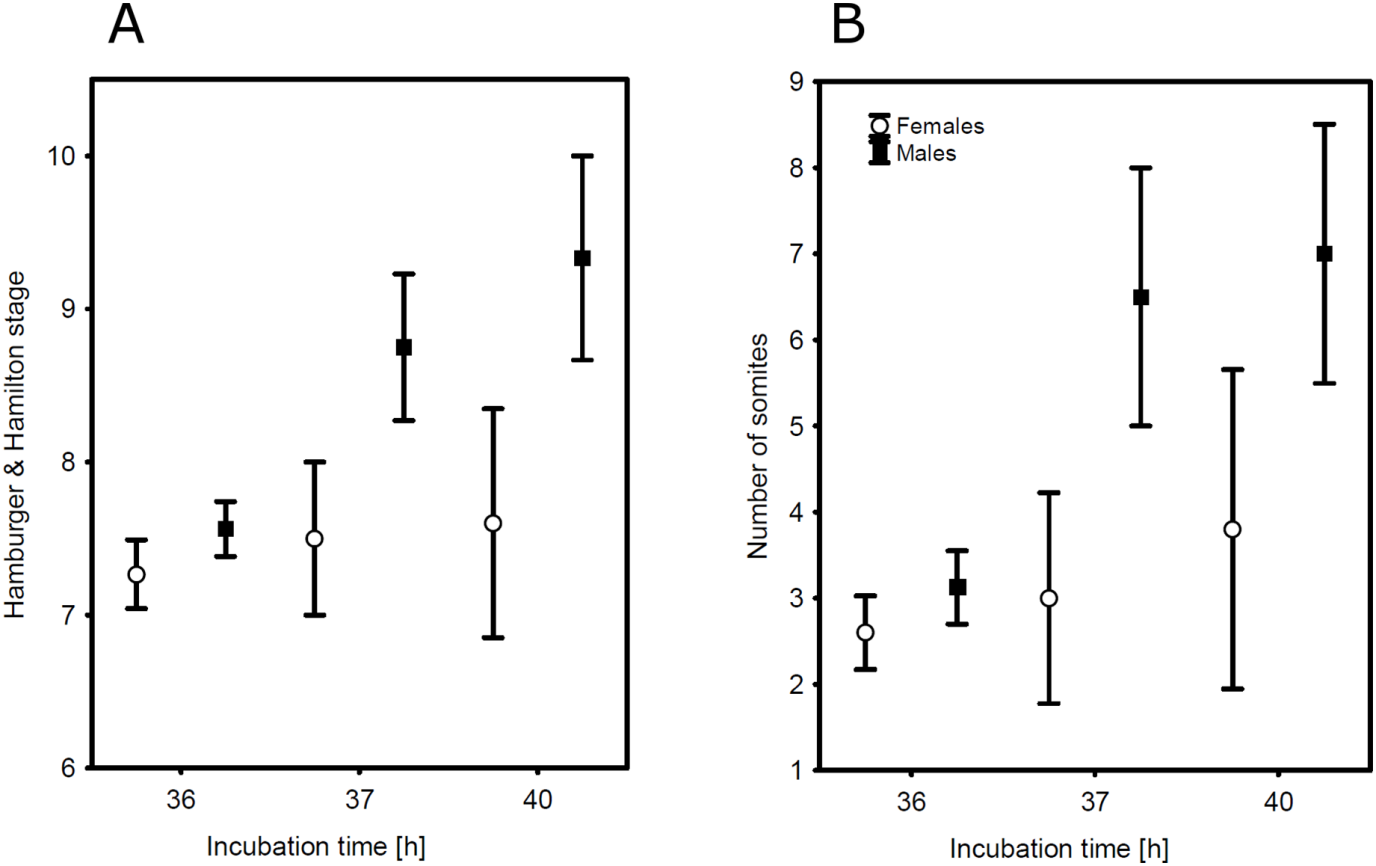
Sexual dimorphism in the growth rate of zebra finch embryos arises after 36 h of incubation. A – Hamburger & Hamilton stage, B – number of somites in male and female embryos in relation to incubation time. Sample sizes for female estimates are 30, 4 and 5 and for male estimates 32, 4 and 6 embryos for incubation of 36, 37 and 40 hours respectively. Mean ± SE are presented.

Analyses controlling for the exact time of the incubation revealed that, in terms of H&H, male embryos were developing faster than female embryos (least square means ± SE after 36 h: males 7.5±0.2, females 7.2±0.2; after 37 h: males 8.8±0.6 females 7.3±0.6; after 40 h: males 9.3±0.5 females 7.5±0.5; sex: F_1, 59_ = 5.50, p = 0.0224, incubation time: F_2, 59_ = 4.43, p = 0.0161, variance explained by the female ID: 12.7%; Fig. 2A;) and that the number of somites in males was also significantly higher than in females (least square means ± SE after 36 h: males 3.1±0.5, females 2.6±0.5; after 37 h: males 6.6±1.3 females 2.4±1.3; after 40 h: males 6.9±1.1 females 3.6±1.1; sex: F_1, 59_ = 4.67, p = 0.0347, incubation time: F_2, 59_ = 5.05, p = 0.0094, variance explained by the female ID 14.4%, Fig. 2B).

### Sex dimorphism in the gene expression level after 36h of incubation

Out of 15 studied genes, four showed significant overexpression in male embryos (Table 2). These genes are involved in cycle control (*SMAD2* and *CDK7*), energy metabolism (*FBP1*) and growth hormone reception (*GHR*). The regulator of GHR on the cell surface (*JAK2*) also had higher expression in male embryos, but the difference was not statistically significant. *SMAD2*, *GHR*, *FBP1* and *JAK2* are located on the Z chromosome. *CDK7* is of unknown location in zebra finches, but it is mapped on Z in chickens [5].

**Table 2:** Summary of analyses of the relative gene expression levels in male versus female 36 h zebra finch embryos.

Genesymbol	Genelocation	Relative gene expression,male/female	Standarderror	95% confidenceintervals	P
***FBP1***	**Z**	**1.928**	**1.317–3.229**	**0.882–4.970**	**0.021**
***SMAD2***	**Z**	**1.651**	**1.319–1.996**	**1.152–2.436**	**0.007**
***CDK7***	**Un**	**1.646**	**1.203–2.520**	**0.733–3.503**	**0.043**
*JAK2*	Z	1.583	1.100–2.797	0.663–3.232	0.108
***GHR***	**Z**	**1.545**	**1.142–2.132**	**0.895–2.742**	**0.045**
*GH1*	27	1.348	1.010–1.833	0.844–2.120	0.223
*PRKAA1*	Z	1.236	0.946–1.603	0.924–1.983	0.124
*NR3C2*	4	1.155	0.680–2.072	0.471–2.243	0.562
*P21*	4A	1.110	0.889–1.353	0.737–1.546	0.360
*MAPK12*	1A	0.936	0.659–1.308	0.530–1.697	0.698
*H6PD*	21	0.864	0.597–1.256	0.521–1.652	0.395
*E2F6*	3	0.857	0.548–1.304	0.436–1.738	0.434
*NR3C1*	13	0.848	0.555–1.285	0.397–1.642	0.436
*CCND1*	5	0.841	0.650–1.164	0.529–1.564	0.316
*CDK1*	6	0.793	0.625–1.050	0.530–1.170	0.129
*NR5A1*	17	detected only in female embryos			

Genes are arranged according to the male/female ratio. Significant deviation from 1∶1 are in bold.

**Figure 3:**
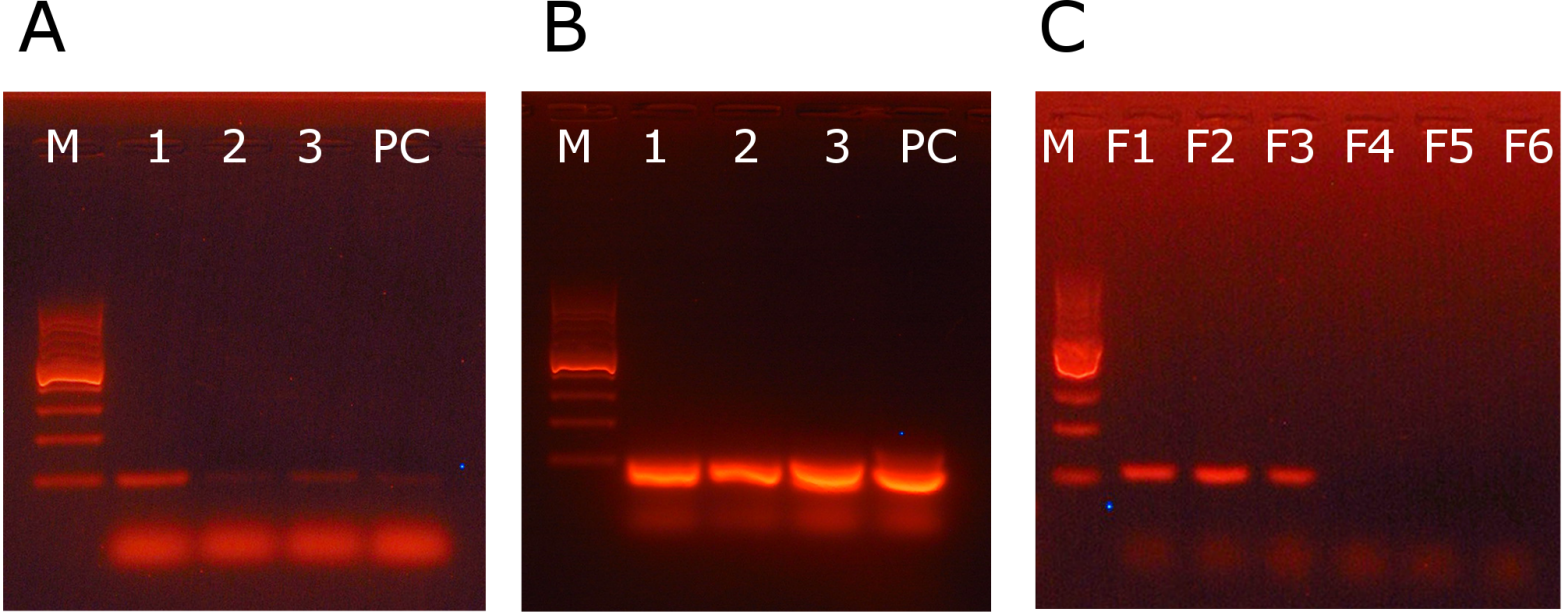
Agarose gel electrophoresis of the PCR amplification products of the *GH1* and *NR5A1* genes. A – expression of the *GH1* gene in the 6 h zebra finch embryos. B – amplification of the reaction product in the nested PCR. Lanes: 1, 2, 3–6 h embryo samples, PC- positive control, reverse transcribed RNA from adult zebra finch pituitary gland. The negative control was RNA treated in the absence of reverse transcriptase. The negative control is not shown, but in all repeats it showed no products. C – expression of the steroidogenic factor 1 gene (*NR5A1*) in female (Lanes: F1, F2 and F3) and lack of the product in male (Lanes: M1, M2 and M3) zebra finch embryos incubated for 36 h. On all photos M is a DNA marker (100–1000 b.p.).

Growth hormone gene (*GH1*) showed similar expression in male and female embryos. Because of the size differences between male and female embryos observed at 36 h of incubation, it was reasonable to expect that expression of the *GH1* gene occurs earlier in the embryonic development. Thus, we analyzed amplification of the *GH1* gene in 3 embryos incubated for 6 h. Expression of the GH1 gene was detected in all the samples and the size of the amplified fragment was 98 b.p. (Fig. 3A, B).

All the genes mapped on the zebra finch autosomes showed similar expression in male and female embryos (Table 2). The exception is *NR5A1* which was expressed uniquely in the females (Fig. 3C).

## Discussion

Our study provides the first evidence of sex differences in growth rate in avian embryos several days before the start of gonad differentiation. Specifically, after 36 hours of incubation, male zebra finches start to develope faster than females (**Fig.** 2). In the presence of equal levels of the growth hormone itself, the faster growth of male embryos is most likely achieved by the overexpression of the growth hormone receptor gene located on the Z chromosome (Table 2). We also found a number of other Z-located genes involved in cell cycle regulation to be differentially expressed in male and female embryos.

Growth hormone (GH) and its receptor (GHR) are the main factors triggering the expression of genes involved in anabolic processes, including protein synthesis, lipid degradation and muscle mass gain [30]. All cells of the body contain GHRs. Its activity is controlled by the kinase JAK2, which stabilizes GHR on the cell surface. In the absence of JAK2, the GHR is rapidly degraded [31]. During postnatal development, tissue growth is regulated by the endocrine GH and the predominant site of its secretion is the anterior pituitary gland, but the early embryonic growth is thought to be regulated by local GH that acts as an autocrine/paracrine factor [32]. Previous studies reported presence of GH mRNA in 2-day-old whole chicken embryos, but in late embryos, the expression of the gene is restricted to specific tissues and cells [32]. The study addressing potential differences between males and females performed on 4-day old embryos reported equal values for the two sexes [33]. Here we found that expression of the growth hormone gene is detectable in the embryos as early as after 6 h of incubation. We conclude that it must have acted on its receptor and thus differentiated male and female growth. Apparently, autocrine/paracrine expression of the *GH1* is intrinsic to the bird embryos from the very beginning of development. We also found that GH shows similar levels in male and female embryos after 36 h of incubation. Thus, the differences in growth rate between males and females are achieved via the differential sensitivity of the cells to the growth factor. To our knowledge, the expression of the receptor of the growth hormone has not been studied before. However, given its location on the Z-chromosome, the overexpression of this gene in male compared to female embryos could be expected due to lack of dosage compensation of several regions of Z chromosome, e.g. [5]. Indeed, we found higher expression of the growth hormone receptor in male embryos incubated for 36 h. The gene *JAK2* has also not been studied before. Because its overexpression in male embryos did not appeared to be statistically significant, we cannot judge on its role in sex-differences reported in this study.

Our results suggest that sex-differences in growth rate of the embryos are also supported by other genes, which we found to be overexpressed in males. The genes *FBP1*, *SMAD2* and *CDK7* are located on the Z chromosome (note that *CDK7* is of unknown location in zebra finches) and were previously reported to be over-expressed in 23 h old male chicken embryos [5]. Here we confirm the finding in embryos incubated for 36 h. Two of those genes are involved in the cell cycle control. The product of *SMAD2* gene mediates the signal of transforming growth factor and acts as the transcription modulator in cell cycle regulation. Cyclins and cyclin-dependent kinases (CDKs) are the key enzymes regulating cell’s progression through the phases of the cell cycle. Cyclins function as regulators of CDKs. *CDK7*, which we found to be up-regulated in male zebra finches, is a cyclin-dependent kinase known to function in both cell cycle regulation and transcription [34]. This kinase and its cyclin, through the chain of complex interrelations, are involved in cell cycle control and RNA transcription by RNA polymerase II. They are also essential for the cell cycle progression via phosphorylation of CDK1 and CDK2 [35]. It is noteworthy that CDK2AP1, which inhibits the activity of CDK2, was found to be overexpressed in female 4-day chicken embryos [24].

In our dataset, the highest overexpression in male compared to female embryos was found for the *FBP1* gene, which is responsible for formation of glucose or glycogen from non-carbohydrates and it also plays a role in suppressing apoptosis [36]. The product of the other Z-located *PRKAA1* gene, which in our study appeared not to be significantly overexpressed in males although it was in the chicken [5], codes for AMP-activated protein kinase (AMPK) known to regulate the cellular energy homeostasis and, on the organism level, in controlling energy balance and food intake [37]. Both of these genes are also involved in insulin signaling networks activating a mitogen-activated protein kinase pathway, which controls cell growth and differentiation [38]. Comparison of the male/female expression ratios of the *FBP1* and *PRKAA1* genes and other Z-located genes (table 2) demonstrates that the degree of dosage compensation of this chromosome is regulated on the a gene-by-gene level (reviewed in [22], [23]). Our results suggest that patterns of dosage compensation might be also species-specific.

We found that several genes, which could be involved in regulation of embryonic growth rate and are located on autosomal chromosomes, showed similar expression in male and female embryos. The exception is *NR5A1*, which gave no sign of gene expression in 36 h male embryos whereas there were clear bands of the gene expression in females (Fig. 3C). The product of the *NR5A1* gene is steroidogenic factor 1 (SF-1) which regulates steroidogenic enzyme expression and plays a key role in the development of adrenal and gonadal glands. It has been previously demonstrated that in the differentiating gonads of birds the expression of this factor is higher in ovaries compared to testes [39]. In our study, we examined the embryos few days before the primordial gonads are formed. Such an early expression of the gene coding SF-1 and its up-regulation in females compared to males may indicate its pivotal role in the triggering of cascade of events resulting in female sex determination in birds. Our finding that most autosomal genes do not differ in expression between male and female embryos is in accordance with previous studies in birds (e.g. [4], [5], [23]) and is most likely related to the fact that in both sexes they are present in two chromosomal copies. Although Itoh et al. [40] found that four genes overexpressed in female zebra finch embryos were all located on autosomes, to our knowledge there are no systematic studied that would investigate the rules of expression patters in autosomal genes in the early embryos.

Our study provides evidence for cell-autonomous sex determination in non-gonadal tissues as predicted by Arnold et al. [41] and demonstrates the phenotypic consequence of lack of full dosage compensation of majority of genes on the avian Z chromosome [5]. The finding of faster development of zebra finch male embryos corroborate the rule observed previously in mammals [6]–[17]. Given that in the blastocyst stage of mammalian embryos the X chromosome inactivation is incomplete and vast of majority of genes show higher expression in female embryos [2], [19], the molecular mechanisms of faster development of males in comparison to females observed in the two taxa has to be different.

The function of the observed phenomenon in mammals is disputed. One suggestion is that differences in growth rate and metabolic activity between male and female pre-implantation embryos could play a role in adaptive manipulation of the embryos that eventually implant and develop (reviewed in [42]). In birds, the function of the observed faster growth of male embryos is even less clear. Detailed description of embryonic development in zebra finches revealed that it is more varied than in the case of the chicken [27]. These could stem from the fact that in domesticated chicken the influence of maternal effects mediated by egg/yolk size and content might be less pronounced than in the non-domesticated zebra finch. Our study suggests that part of the observed variation in embryo size could also be attributed to its sex. It remains to be confirmed whether sex-specific growth rate is a common phenomenon in birds and whether it has adaptive functions. Currently we can only speculate that differential growth at the early stage could be necessary for development of some sex-specific structures. Given that at hatching male and female zebra finches are monomorphic, it would also be of interest to understand the mechanism and timing of the catch-up growth. Finally, future studies should explore the potential link between the male and female growth trajectories and embryonic viability, especially in the context of abovementioned maternal effects.

In conclusion, we demonstrate sex-specific growth/developmental rate of zebra finch embryos before the onset of gonadogensis. The effect is driven by overexpression of the suite of genes located on the Z chromosome and responsible for growth, metabolism and cell cycle regulation. We suggest that faster growth of early male embryos is conserved through the mammalian and bird phyla, irrespective of their differential sex chromosome systems and differential genetic pathways responsible for the effect. Our findings elucidate patterns of early embryogenesis which may be of fundamental importance for the sex-specific regulation of metabolic pathways and for the fine regulation of offspring sex ratio in birds.
